# Data on the construction of a recombinant HEK293 cell line overexpressing hERG potassium channel and examining the presence of hERG mRNA and protein expression

**DOI:** 10.1016/j.dib.2017.08.008

**Published:** 2017-08-12

**Authors:** Yi Fan Teah, Muhammad Asyraf Abduraman, Azimah Amanah, Mohd Ilham Adenan, Shaida Fariza Sulaiman, Mei Lan Tan

**Affiliations:** a Malaysian Institute of Pharmaceuticals & Nutraceuticals, National Institutes of Biotechnology Malaysia (NIBM), Ministry of Science, Technology and Innovation Malaysia, Pulau Pinang, Malaysia; b Advanced Medical & Dental Institute, Universiti Sains Malaysia, Pulau Pinang, Malaysia; c Atta-ur-Rahman Institute for Natural Product Discovery, Universiti Teknologi MARA (UiTM), Selangor Darul Ehsan, Malaysia; d School of Biological Sciences, Universiti Sains Malaysia, Pulau Pinang, Malaysia

**Keywords:** hERG, Recombinant cell line, hERG mRNA expression, hERG protein expression

## Abstract

The data presented in this article are related to the research article entitled “The effects of deoxyelephantopin on the cardiac delayed rectifier potassium channel current (I_Kr_) and human ether-a-go-go-related gene (hERG) expression” (Y.F. Teah, M.A. Abduraman, A. Amanah, M.I. Adenan, S.F. Sulaiman, M.L. Tan) [1], which the possible hERG blocking properties of deoxyelephantopin were investigated. This article describes the construction of human embryonic kidney 293 (HEK293) cells overexpressing HERG potassium channel and verification of the presence of hERG mRNA and protein expression in this recombinant cell line.

**Specifications Table**

**Table:** 

Subject area	*Pharmacology and Toxicology*
More specific subject area	*Stable transfection, RT-qPCR and Western Blot analysis*
Type of data	*Figures, text file*
How data was acquired	*CFX96™ Real-Time PCR Detection System (Bio-Rad Laboratories, USA), ChemiDoc*^*™*^*XRS Imaging System (Bio-Rad Laboratories, USA)*
Data format	*Analyzed*
Experimental factors	*Transfected HEK293 cells with pCMV6-Neo-hERG plasmid and non-transfected HEK293 cells were analyzed using RT-qPCR and Western Blot analysis*
Experimental features	*The presence of hERG mRNA and protein expression in HEK 293-hERG cell line were determined*
	*The absence of hERG mRNA and protein expression in non-transfected HEK293 cells were observed*
Data source location	*Universiti Sains Malaysia, Pulau Pinang, Malaysia*
	*Malaysian Institute of Pharmaceuticals and Nutraceutical, NIBM, Pulau Pinang, Malaysia*
Data accessibility	*Data is accessible with this article*

**Value of the data**

• The data is beneficial to researchers who are interested in the pharmacology properties of deoxyelephantopin and *Elephantopus scaber* Linn
• This data set is beneficial to researchers who want to construct a heterologous mammalian system expressing hERG potassium channel
• The data is helpful to determine the mRNA and protein expression of hERG in cell lines after stable transfection
• The data is helpful to ensure that the recombinant cell line (HEK293-hERG) is expressing hERG at both transcriptional and translational level.

## 1 Data

**Fig. 1:**
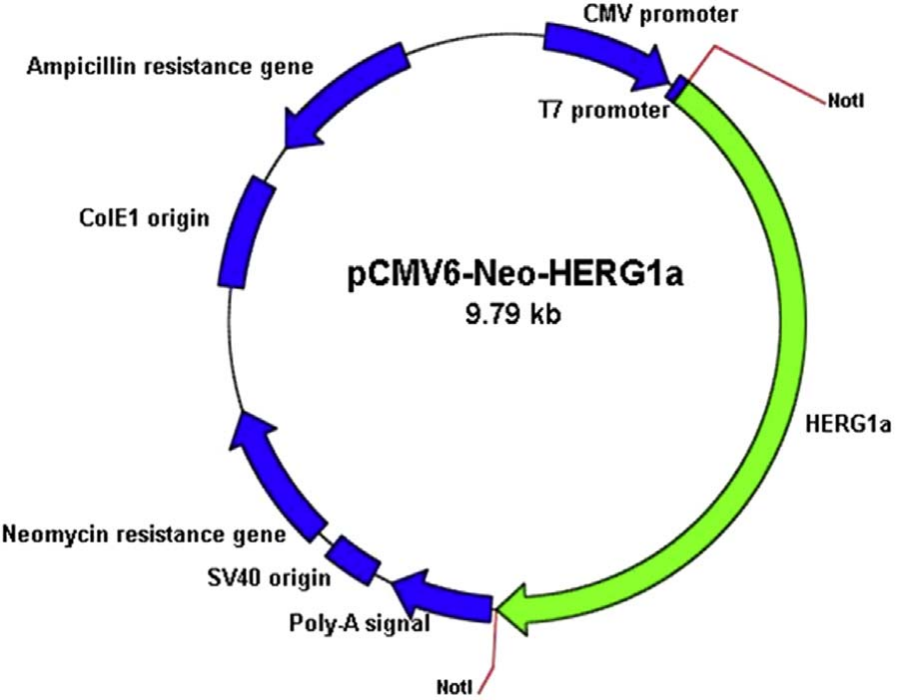
Plasmid map of pCMV6-Neo-hERG (adapted from Origene Technologies, USA).

**Fig. 2:**
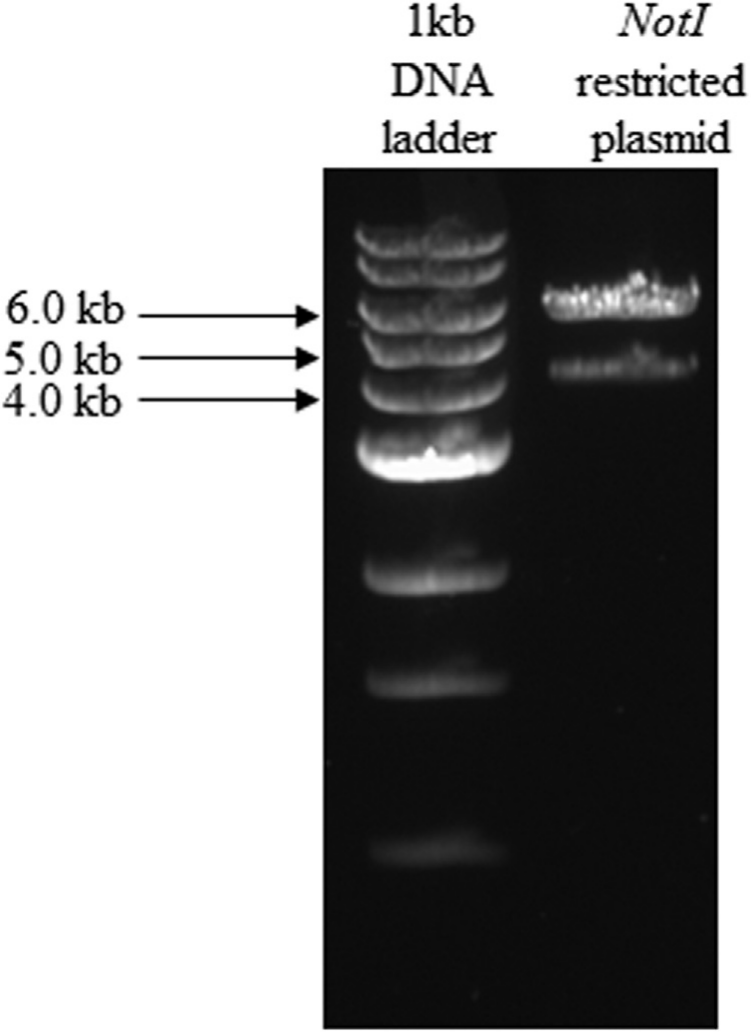
Gel electrophoresis of restriction enzyme digestion products of the plasmid map. Lane 1:1 kb DNA ladder; Lane 2: *NotI* restricted plasmid sample where 6 kb refers to the fragment of plasmid and 4 kb band refers to the hERG cDNA insert.

**Fig. 3:**
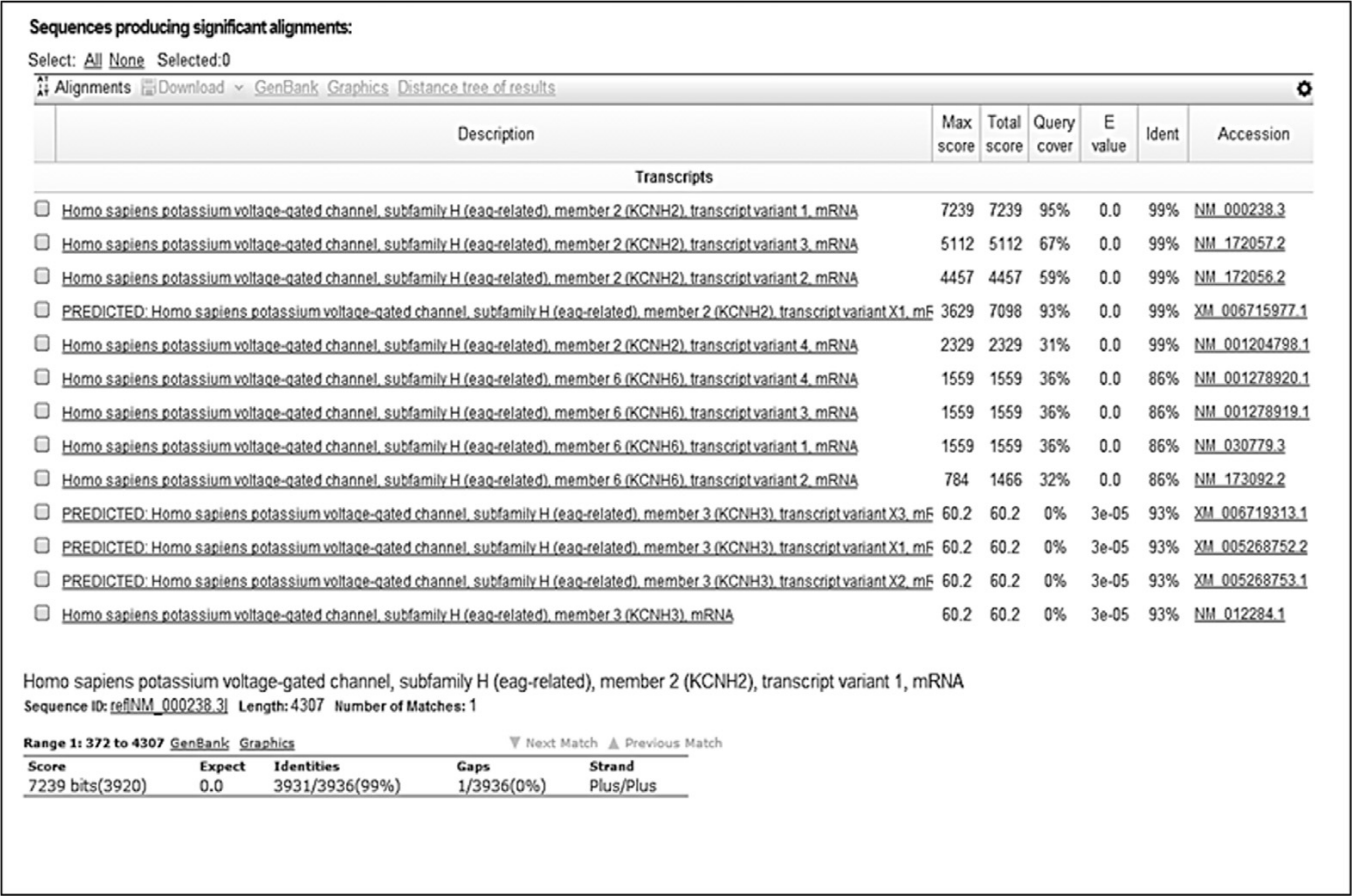
Sequence alignment and comparison between the sequences of the cDNA insert against hERG sequence [NM 000238.3 homo sapiens potassium voltage-gated channel, subfamily H (eag-related), member 2 (KCNH2), transcript variant 1, mRNA].

**Fig. 4:**
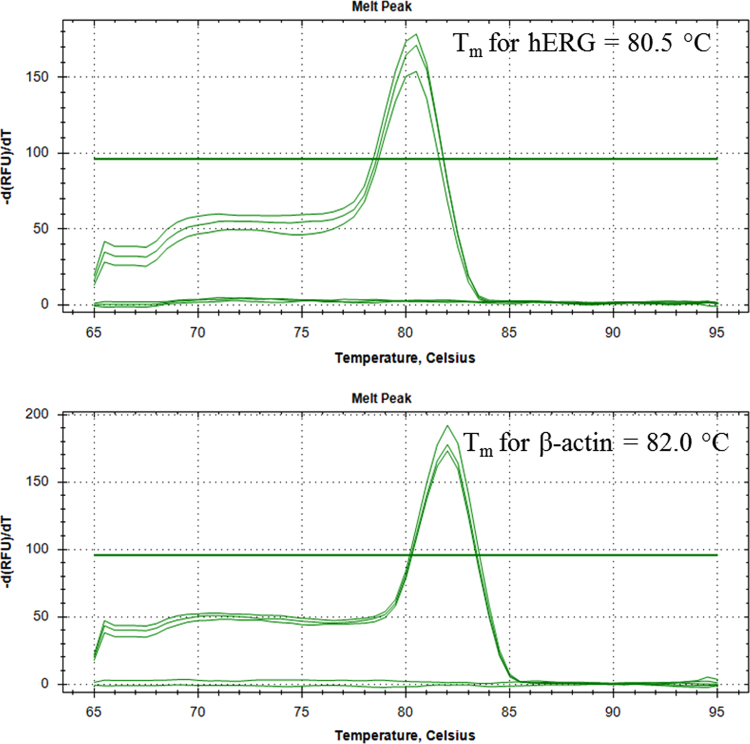
Melt curves displaying the melting temperature (T_m_) as peaks. The T_m_ for hERG expression was 80.5 °C and the T_m_ for β-actin expression was 82.0 °C.

**Fig. 5:**
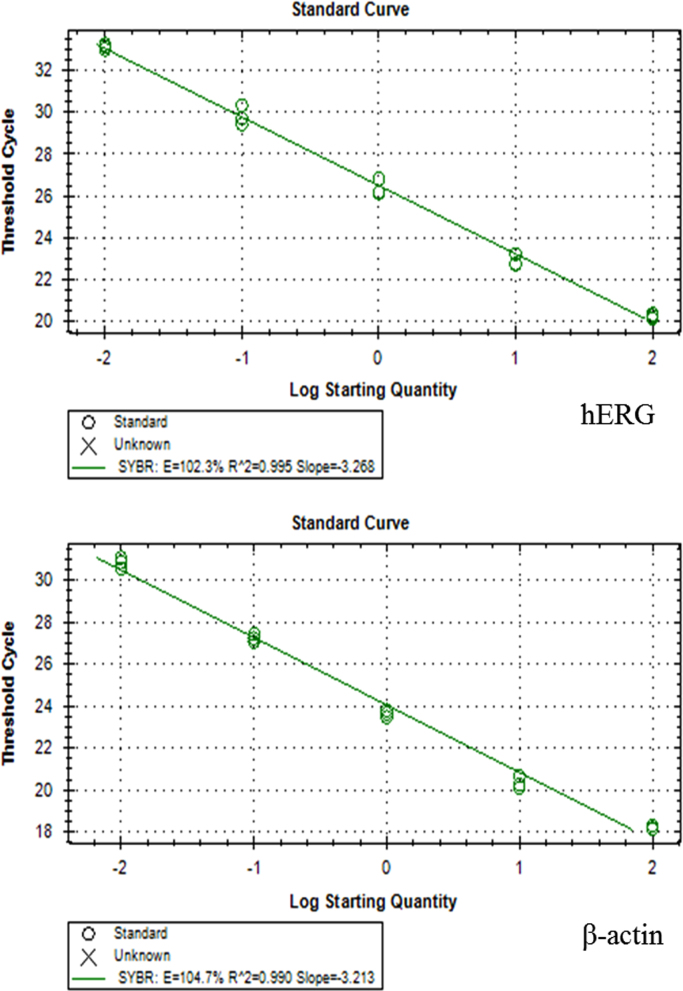
Standard curve plots for amplification efficiency of hERG and β-actin. PCR efficiency (E) was determined using CT slope method with data points covering a log dilution range. Data was calculated automatically by CFX Manager software version 3.1 (Bio-Rad Laboratories, USA). The calculated amplification efficiency for hERG was 102.3% and that of β-actin was 104.7%.

**Fig. 6:**
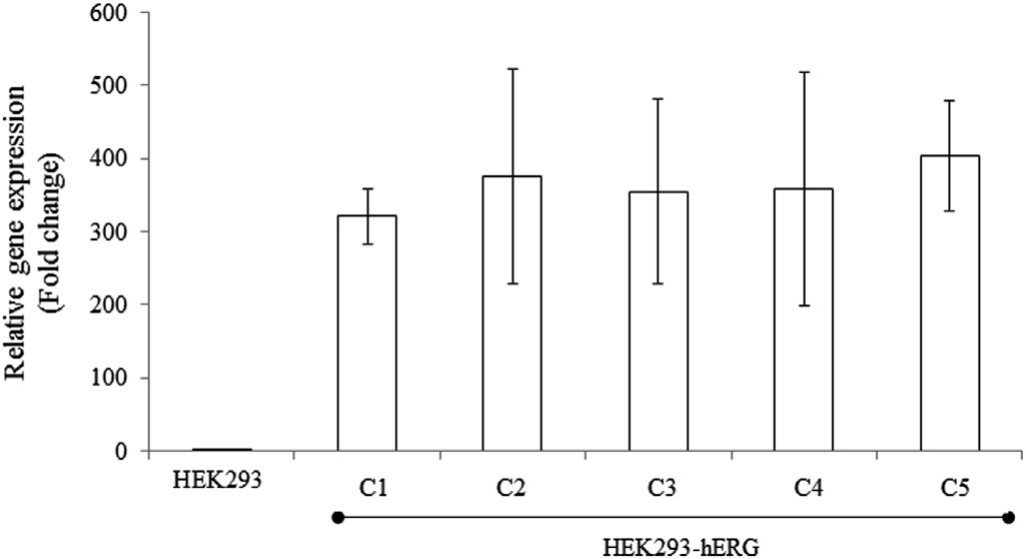
Graphical representation of the hERG mRNA expressions in HEK293 and HEK293-hERG cell line. Data are represented as the means ± SD from 2 independent experiments (n=6).

**Fig. 7:**
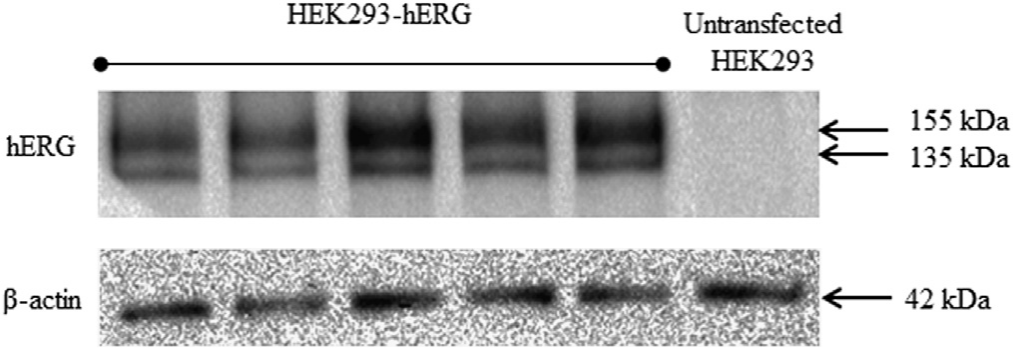
A representative image showing the Western blot analysis of the hERG protein expression in hERG-transfected and non-transfected HEK293 cells.

Fig. 1 shows the plasmid map of pCMV6-Neo-hERG (Origene, USA). Fig. 2 shows the restriction enzyme digestion products of the plasmid. Fig. 3 shows the sequence alignment and comparison between the sequences of the cDNA insert against the hERG sequence [NM 000238.3 homo sapiens potassium voltage-gated channel, subfamily H (eag-related), member 2 (KCNH2), transcript variant 1, mRNA]. Fig. 4 shows the melt curves displaying the melting temperature (T_m_) as single peaks. Fig. 5 shows the standard curve plots for amplification efficiency for hERG and β-actin. Fig. 6 shows significant hERG mRNA expressions in different batches of HEK293-hERG cells. Fig. 7 shows a representative image of the hERG channel protein expression in hERG-transfected and non-transfected HEK293 cells [1].

## 2 Experimental design, materials and methods

### 2.1 Cell culture and plasmid amplification

HEK293 cell line was purchased from American Type Culture Collection (ATCC, USA). Cells were maintained in Dulbecco's Modified Eagle medium (DMEM) supplemented with 10% (v/v) fetal bovine serum (FBS), 100 U/ml penicillin, 100 µg/ml of streptomycin, 1% (v/v) sodium pyruvate, 1% (v/v) sodium bicarbonate and 1% (v/v) Minimum Essential Medium (MEM) non-essential amino acid. Cultures were incubated at 37 °C in a humidified incubator supplemented with 5% (v/v) CO_2_. Mammalian expression vector carrying hERG cDNA (pCMV6-Neo-hERG) was purchased from OriGene Technologies (USA). The recombinant plasmid map is illustrated in Fig. 1. Amplification of this plasmid DNA was carried out by transformation into the DH5α strain of *Escherichia coli* bacteria (Invitrogen, USA) using standard laboratory protocol. Extraction of plasmid DNA was carried out using Plasmid Midi kit (Qiagen, USA). Digestion of the plasmid was carried out using restriction enzyme *NotI* (New England Biolabs, USA), where the restriction sites were indicated as illustrated in Fig. 1. Gel electrophoresis was performed with 0.7% (w/v) agarose gel to confirm the size of plasmid fragments after restriction enzyme digestion. The recombinant plasmid, pCMV6-Neo-hERG was sent for DNA sequencing at Macrogen (Seoul, Korea). The obtained sequence was then analyzed using Nucleotide Basic Local Alignment Searching Tool (BLAST) from National Center for Biotechnology Information (NCBI) (http://blast.ncbi.nlm.nih.gov/).

### 2.2 Stable transfection

HEK293 cells were suspended in an appropriate volume of ice-cold Opti-MEM® (Gibco, USA) electroporation buffer. Cell suspension containing approximately 2.5 × 10^5^ cells were aliquoted into pre-chilled electroporation cuvette. An aliquot containing 2.5 µg of pCMV6-Neo-hERG clone (OriGene Technologies, USA) was added into the cuvette. Sterile deionized water added into cell suspension was used as blank electroporation control. The cuvette was then inserted into the Gene Pulser Xcell™ electroporation system (Bio-Rad Laboratories, USA) and electroporation was carried out using single pulse at 240 V and for 25 ms. The cells were then transferred into a T25 flask containing 5 mL of complete medium and incubated at 37 °C for 48 h. After 48-h maintenance in complete medium without selection antibiotic, both sets of cells (with cDNA clone and blank control) were supplemented with complete medium containing Geneticin^®^ in increasing concentration over several weeks. Surviving cells cultured in complete medium containing 500 µg/mL of G418 sulfate were selected manually using a pipette tip and transferred into 24-well plates. These cells were grown separately and propagated to ensure monoclonal populations of recombinant cells were obtained.

### 2.3 Verification of hERG mRNA expression

The mRNA expression of hERG gene in selected stably transfected cells (HEK293-hERG) and the non-transfected HEK293 cells were determined using RT-qPCR. Total RNA was isolated using QIAshredder^™^ and RNeasy^®^ Mini Kit according to the manufacturer's instructions. Quantification and purity of RNA samples was determined using Ultrospec™ 3100 *pro* UV/Visible spectrophotometer (Amersham Biosciences, U.K.). Primers were designed using Beacon Designer software, Version 7.7 (Premier Biosoft International, USA) based on sequence data obtained for hERG (NM_000238.3) and β-actin (NM_001101.3) from the National Centre of Biotechnology Information (NCBI) database (http://www.ncbi.nlm.nih.gov/) (Table 1). RT-qPCR was carried out using Bio-Rad^®^ CFX^™^ real time PCR system and iScript^™^ One-Step RT-PCR kit with SYBR^®^ Green (Bio-Rad Laboratories, USA). Briefly, the RT-qPCR amplification was carried out in final volume of 25 µL according to manufacturer's protocol and program as shown in Table 1. The hERG mRNA expression was determined by analyzing the threshold cycle (C_t_) value for each RNA sample extracted from both transfected HEK293-HERG cells and non-transfected HEK293 cells (control). The data were presented as fold change in gene expression normalized to an endogenous reference β-actin gene and relative to the non-transfected control.

**Table 1:** Primer sequence, RT-PCR composition and thermal cycler program.

**Primers**					
Gene accession no.	Sequence			Amplicon size (bp)	Optimized annealing temperature (°C)
hERG (NM_000238.3)	F	5′-AAATCACCCTCAACTTTG-3′		96	53
	R	5′-TTCGCTCCTTTATCTTAG-3′			
β-actin (NM_001101.3)	F	5′- ATCACCATTGGCAATGAG-3′		105	
	R	5′-GATGGAGTTGAAGGTAGTT-3′			
			
**Reaction Mixture**					
Reagent			Final concentration		Volume (μl)
2 X SYBR Green RT-PCR Reaction Mix			1 X		12.5
10 µM forward primer			300 nM		0.75
10 µM reverse primer			300 nM		0.75
Nuclease-free water			-		9.5
iScript™ Reverse Transcriptase			-		0.5
RNA template			100 ng		1
Total volume					25
		
**Program**					
RT-PCR cycle			Steps		Temperature and duration
Reverse transcription			cDNA synthesis		50.0 °C for 10 min
			Inactivation of reverse transcriptase		95.0 °C for 5 min
PCR cycling and detection (40 cycles)			Denaturation		95.0 °C for 10 s
			Annealing & extension		Gradient for 30 s
			Melt curve analysis (60 cycles)		65 °C to 95 °C:
					Increment 0.5 °C for 5 s
			Hold		10.0 °C ∞

### 2.4 Verification of hERG protein expression

Total protein was isolated using Mem-PER™ Plus membrane protein extraction kit (ThermoScientific, USA) and was purified using Pierce^®^ SDS-PAGE Sample Prep Kit (ThermoScientific, USA) according to the manufacturer's protocol. Protein concentration was measured using Biorad D_C_ Protein Assay and Protein Standard II BSA (Bio-Rad Laboratories, USA). Total membrane protein was separated using SDS-PAGE with 4% (v/v) stacking gel and 10% (v/v) resolving gel. Briefly, fractionated proteins were first transferred to Immobilon-P PVDF membrane (Milipore, USA) and blocked with 3% (w/v) skim milk for 1 h and incubated with optimized primary antibodies against hERG [1:250; Santa-Cruz Biotechnology (USA)] and β-actin [1:1000; Cell Signaling Technology (USA)] overnight at 4°C. The membrane was then incubated in horseradish peroxidase conjugated secondary antibodies [Cell Signaling Technology (USA)] for 2.5 h followed by ECL^™^ Western Blotting Detection Reagents (GE Healthcare, UK). Chemiluminescence signal was analyzed using ChemiDoc^™^ XRS Imaging System (BioRad Laboratories, USA).
